# Atypical Bovine Spongiform Encephalopathies, France, 2001–2007

**DOI:** 10.3201/eid1402.071141

**Published:** 2008-02

**Authors:** Anne-Gaëlle Biacabe, Eric Morignat, Johann Vulin, Didier Calavas, Thierry G.M. Baron

**Affiliations:** * Agence Française de Sécurité Sanitaire des Aliments, Lyon, France

**Keywords:** BSE, H-type, L-type, BASE, prion, cattle, dispatch

## Abstract

In France, through exhaustive active surveillance, ≈17.1 million adult cattle were tested for bovine spongiform encephalopathy from July 2001 through July 2007; ≈3.6 million were >8 years of age. Our retrospective Western blot study of all 645 confirmed cases found that 7 were H-type and 6 were L-type.

Most cases of bovine spongiform encephalopathy (BSE) have shown strikingly uniform features. The origin of epidemics, mainly in the United Kingdom but to a lesser extent in other countries, has been foodborne contamination by a single major strain of the transmissible spongiform encephalopathy (TSE) agent (*1**,**2*). However, in recent years, 2 distinct forms of the disease have been described; these forms deviate phenotypically from the previously identified classic BSE (C-type) (*3**,**4*). Western blot studies of the protease-resistant prion protein (PrPres) showed higher and lower molecular masses of unglycosylated PrPres in these 2 types, subsequently named H-type and L-type compared with C-type BSE (*5*). In L-type BSE, the most discriminant molecular feature was the lower level of diglycosylated PrPres (*4**,**5*). Such cases have now been identified in a number of different countries (*6**–**8*).

The origin of H-type and L-type BSE cases is unknown, but they may represent spontaneous or so-called sporadic forms of TSE, reminiscent of most cases of Creutzfeldt-Jakob disease in humans. Transmission studies in wild-type mice (*9*) or in transgenic mice expressing the bovine PrP gene (*5**,**10**–**12*) have shown that the infectious agents involved in H-type and L-type BSEs differ from the single strain isolated from C-type BSE.

Epidemiologic data are crucial to our understanding of the origin of such cases, but precise prevalence needs to be determined. The prevalence in cattle can now be assessed with accuracy because exhaustive BSE testing of adult cattle was implemented in 2001 in all European member states (January 1 for abattoirs and July 1 for rendering plants). We report the results of a retrospective study to determine the frequency of H-type and L-type BSE identified in France since July 2001.

## The Study

A retrospective Western blot study was performed for all confirmed BSE cases diagnosed in France after July 1, 2001, as previously described (*13*). Briefly, PrPres was extracted using the TeSeE Western blot confirmatory assay (Bio-Rad, Marnes-la-Coquette, France) according to the manufacturer’s instructions. For immunoblotting, antibodies RB1, 6H4 (R-Biopharm, St. Didier au Mont d’Or, France), Sha31 from the TeSeE Western blot (Bio-Rad), and SAF84 (kindly provided by J. Grassi, Commissariat Energie Atomique, Saclay, France) were used; these antibodies recognize the bovine PrP sequences 110–113, 156–164, 156–163, and 175–180, respectively. Two Western blot assays were conducted on the brainstem samples and used either an antibody against the PrP core (RB1, 6H4, or Sha31) or a C-terminal antibody (SAF84) (*13*). The characteristics of H-type and L-type BSE (*6**,**13*) that were sought included 1) a higher or lower molecular mass of unglycosylated PrPres with core antibodies in H-type and L-type BSE compared with C-type BSE, 2) a lower proportion of diglycosylated PrPres in L-type BSE, and 3) presence of an additional band at ≈14 kDa with SAF84 in H-type BSE. All cases with features of H-type or L-type BSE were then subjected to further Western blot analyses for detailed quantitative analysis of PrPres molecular features (apparent molecular masses and glycoforms proportions).

Among the 645 BSE cases confirmed between July 1, 2001, and July 1, 2007, 7 H-type and 6 L-type isolates were identified; these had occurred at a frequency of 0–3 H-type and 1 L-type case per year, compared with 6–219 cases of C-type BSE per year (Table). Molecular typing of 48 of these 645 samples was not possible because low levels of PrPres prevented detailed molecular characterization or because sample amount was insufficient. All 13 atypical cases were detected in cattle >8 years of age, from fallen stock (9 cases) or abattoir (4 cases); not 1 atypical case was found among the 98 BSE cases detected by clinical surveillance during this period. These 13 atypical cases were diagnosed by the different rapid BSE tests routinely used in France: 9 by Prionics-Check Western, LIA, or Priostrip (AES, Combourg, France), 3 by ELISA Bio-Rad, and 1 by IDEXX HerdChek (IDEXX Laboratories, Schiphol-Rijk, the Netherlands). During retrospective interviews, the farmer and veterinarian for 6 of these animals reported clinical signs consistent with TSE in 3 fallen stock. This series of 13 cases identified since the beginning of exhaustive active surveillance should be compared with a total of ≈17.1 million adult cattle tested, of which ≈3.6 million were >8 years of age. In addition to these 13 cases, the first case of atypical (H-type) BSE was identified in 2000, during an exhaustive active surveillance program in rendering plants in a limited region of France.

**Table:** Results of retrospective molecular typing studies for types of bovine spongiform encephalopathy (BSE) cases identified, France, 2001–2007*

Year	No. tested	Age >8 y	H-BSE	L-BSE	C-BSE	UC
2001 (Jul–Dec)	1,524,344	334,865	1	0	153	18
2002	3,183,320	648,026	2	1	219	17
2003	3,189,899	649,724	3	1	123	10
2004	2,867,571	614,851	0	1	51	2
2005	2,590,973	565,863	0	1	30	1
2006	2,692,048	555,577	0	1	6	0
2007 (Jan–Jun)	1,070,210	244,286	1	1	2	0
Total	17,118,365	3,613,192	7	6	584	48

*H-type, higher molecular masses of unglycosylated protease-resistant prion protein (PrPres); L-type, lower molecular masses of unglycosylated PrPres; C-type, classic BSE; UC, unclassified.

**Figure 1:**
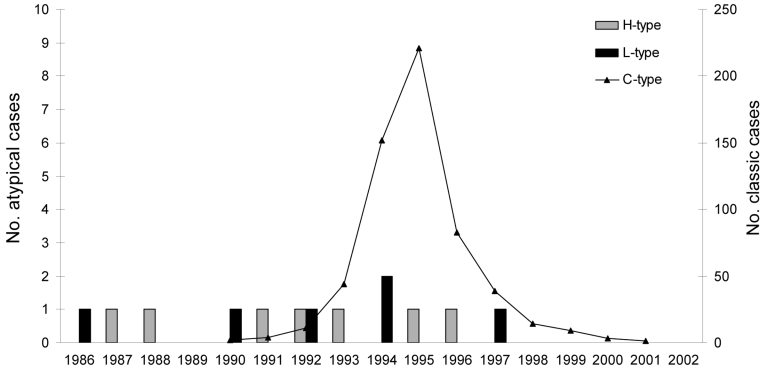
Distribution of bovine spongiform encephalopathy cases identified from July 1, 2001, through July 1, 2007, by year of cattle birth. H-type, higher molecular masses of unglycosylated protease-resistant prion protein (PrPres); L-type, lower molecular masses of unglycosylated PrPres; C-type, classic BSE.

**Figure 2:**
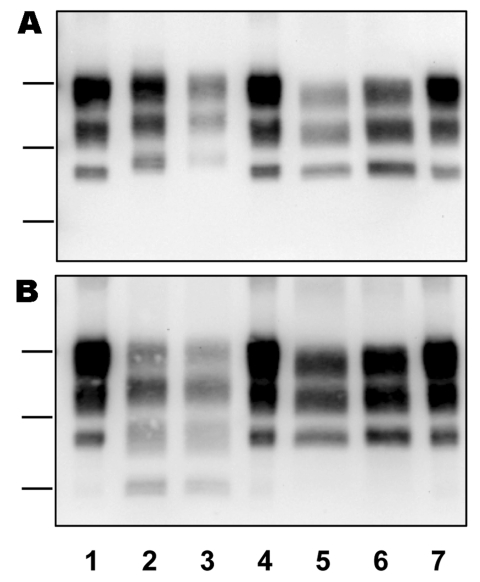
Representative Western blot analyses of protease-resistant prion protein (PrPres) in H-type (lanes 2, 3), L-type (lanes 5, 6), and C-type (lanes 1, 4, 7) cases of bovine spongiform encephalopathy (BSE). Bars to the left of the panels indicate the 29.0-, 20.1-, and 14.3-kDa marker positions. H-type, higher molecular masses of unglycosylated PrPres; L-type, lower molecular masses of unglycosylated PrPres; C-type, classic BSE. Monoclonal antibodies Sha31 and SAF84 were used for PrPres detection in panels A and B, respectively.

The distribution of BSE-infected cattle by birth date (Figure 1) shows that 1 or 2 (H- or L-type cases) were eventually found positive by rapid tests of the brainstem in each annual birth cohort from 1986 through 1997, which compares with up to 221 cattle infected with C-type BSE born during 1990–2001 for which BSE was diagnosed during 2001–2007. All H-type and L-type BSE cases showed similar features (Figure 2) regarding the high apparent molecular masses of unglycosylated PrPres (mean difference of ≈1 kDa using 6H4 antibody) for H-type BSE and lower levels of diglycosylated PrPres (mean difference of 35% using 6H4 antibody) for L-type BSE, compared with C-type BSE. For L-type BSE cases, the differences in apparent molecular masses were more obvious for the diglycosylated band (≈1-kDa difference using 6H4, compared with 0.3 kDa for the unglycosylated band), as previously observed (*6*). The ≈14-kDa band characteristic of H-type BSE was similarly detected in the 8 isolates with SAF84 antibody but in none of the other cases classified as L-type or C-type BSE.

## Conclusions

This study involved exhaustive molecular typing of BSE cases during a given test period and in a country in which BSE testing has been mandatory for all adult slaughtered or fallen cattle. France tests more animals than any other European country; ≈30% of the animals tested in the European Union are tested in France. The estimated frequency of H-type and L-type BSE was 0.41 and 0.35 per million adult cattle tested, respectively (1.9 and 1.7 in cattle >8 years of age). Given the implementation dates of measures to control BSE and the birth dates of these BSE-infected cattle, foodborne exposure to an infectious agent cannot be fully excluded for any of these cattle. However, the distribution of cattle affected by H- and L-type BSE, by year of birth, differs strikingly from that of cattle affected by C-type BSE and is consistent with the hypothesis that H-type and L-type BSE might represent sporadic prion disorders. In comparison with another sporadic prion disease, the annual frequency of sporadic Creutzfeldt-Jakob disease in humans, which is estimated only by analyses of reported clinically suspect cases, is 1–2 cases per million. Similar studies in other countries, instead of those free of C-type BSE, would be useful. An alternative hypothesis to foodborne contamination, such as contamination by a scrapie agent, cannot be fully excluded, as such contamination has been shown to be a risk factor for scrapie in sheep (*14*).

This study relied on the identification of BSE cases by examination of the brainstem only, as derived from our knowledge of C-type BSE (*15*). We cannot exclude possible differences in the pathogenesis of atypical BSEs that might result in underestimation of their frequency, e.g., involvement of the brainstem at a later stage than with C-type BSE. This possibility is at least suggested by data available for L-type BSE, which shows a preferential distribution of abnormal PrP in more rostral brain regions (*4**,**12*). Studies of the pathogenesis of these novel BSE forms are thus important for understanding of prion disorders of domestic ruminant species.

## References

[1] Simmons MM, Harris P, Jeffrey M, Meek SC, Blamire IW, Wells GA. BSE in Great Britain: consistency of the neurohistopathological findings in two random annual samples of clinically suspect cases. Vet Rec. 1996;138:175–07. 8677617 10.1136/vr.138.8.175

[2] Fraser H, Bruce ME, Chree A, McConnell I, Wells GA. Transmission of bovine spongiform encephalopathy and scrapie to mice. J Gen Virol. 1992;73:1891–7. 10.1099/0022-1317-73-8-1891 1645134

[3] Biacabe AG, Laplanche JL, Ryder S, Baron T. Distinct molecular phenotypes in bovine prion diseases. EMBO Rep. 2004;5:110–5. 10.1038/sj.embor.7400054 14710195 PMC1298965

[4] Casalone C, Zanusso G, Acutis P, Ferrari S, Capucci L, Tagliavini F, Identification of a second bovine amyloidotic spongiform encephalopathy: molecular similarities with sporadic Creutzfeldt-Jakob disease. Proc Natl Acad Sci U S A. 2004;101:3065–70. 10.1073/pnas.0305777101 14970340 PMC365745

[5] Buschmann A, Gretzschel A, Biacabe AG, Schiebel K, Corona C, Hoffmann C, Atypical BSE in Germany—proof of transmissibility and biochemical characterization. Vet Microbiol. 2006;117:103–16. 10.1016/j.vetmic.2006.06.016 16916588

[6] Jacobs JG, Langeveld JPM, Biacabe AG, Acutis P, Polak MP, Gavier-Widen D, Molecular discrimination of atypical bovine spongiform encephalopathies from a wide geographical region in Europe. J Clin Microbiol. 2007;45:1821–9. 10.1128/JCM.00160-07 17442800 PMC1933055

[7] Richt JA, Kunkle RA, Alt D, Nicholson EM, Hamir AN, Czub S, Identification and characterization of two bovine spongiform encephalopathy cases diagnosed in the United States. J Vet Diagn Invest. 2007;19:142–54. 17402608 10.1177/104063870701900202

[8] Hagiwara K, Yamakawa Y, Sato Y, Nakamura Y, Tobiume M, Shinagawa M, Accumulation of mono-glycosylated form-rich, plaque-forming PrP^Sc^ in the second atypical bovine spongiform encephalopathy case in Japan. Jpn J Infect Dis. 2007;60:305–8. 17881874

[9] Baron TG, Biacabe AG, Bencsik A, Langeveld JP. Transmission of new bovine prion to mice. Emerg Infect Dis. 2006;12:1125–8. 16836832 10.3201/eid1207.060107 PMC3291063

[10] Béringue V, Bencsik A, Le Dur A, Reine F, Laï TL, Chenais N, Isolation from cattle of a prion strain distinct from that causing bovine spongiform encephalopathy. PLoS Pathog. 2006;2:e112. 10.1371/journal.ppat.0020112 17054396 PMC1617128

[11] Béringue V, Andréoletti O, Le Dur A, Essalmani R, Vilotte JL, Lacroux C, A bovine prion acquires an epidemic BSE strain-like phenotype upon interspecies transmission. J Neurosci. 2007;27:6965–71. 10.1523/JNEUROSCI.0693-07.2007 17596445 PMC6672218

[12] Capobianco R, Casalone C, Suardi S, Mangieri M, Miccolo C, Limido L, Conversion of the BASE prion strain into the BSE strain: the origin of BSE? PLoS Pathog. 2007;3:e31. 10.1371/journal.ppat.0030031 17352534 PMC1817656

[13] Biacabe AG, Jacobs JG, Bencsik A, Langeveld JPM, Baron T. H-type bovine spongiform encephalopathy: complex molecular features and similarities with human prion diseases. Prion. 2007;1:61–8 [cited 2007 Dec 13]. Available from http://www.landesbioscience.com/journals/prion/) 10.4161/pri.1.1.3828 PMC2633710 19164888

[14] Philippe S, Ducrot C, Roy P, Remontet L, Jarrige N, Calavas D. Sheep feed and scrapie, France. Emerg Infect Dis. 2005;11:1274–9. 16102318 10.3201/eid1108.041223 PMC3320489

[15] Grassi J, Comoy E, Simon S, Creminon C, Frobert Y, Trapmann S, Rapid test for the preclinical postmortem diagnosis of BSE in central nervous system tissue. Vet Rec. 2001;149:577–82. 11730165 10.1136/vr.149.19.577

